# The Influence of EFL Teachers’ Hope and Trust on Their Academic Grit: A Theoretical Review

**DOI:** 10.3389/fpsyg.2022.929841

**Published:** 2022-06-02

**Authors:** Yushu Xu

**Affiliations:** Department of Foreign Languages, Shanghai University of Finance and Economics Zhejiang College, Jinhua, China

**Keywords:** positive psychology, EFL teachers, hope, trust, academic grit

## Abstract

The role of emotions in second/foreign language education has been exponentially highlighted in the literature. However, the interplay of English as a foreign language (EFL) teachers’ hope, trust, and grit has witness a scant attention among L2 researchers. Against this shortcoming, the present mini-review article made an effort to offer a theoretical analysis of the theoretical and empirical underpinnings of these three constructs. In so doing, it presented the definitions, conceptualizations, dimensions, theories, related studies, and the way these variables can influence one another. Drawing on scientific findings in the literature, this study proposed some implications for EFL teachers, teacher trainers, principals, and scholars to enhance their knowledge of psycho-emotional factors and how establishing an environment based on hope and trust can generate success in L2 education. Finally, some recommendations for future research are made to drive this line of research forward.

## Introduction

Teaching a second/foreign language has been regarded as a tough task due to the existence of a multitude of psycho-emotional variables that considerably influence the success and direction of the teaching-learning cycle (Mercer, 2020; Sikma, 2021). To perform well in such a multi-faceted process, teachers must cope with several factors associated to personality, cognition, cultural differences, context, psycho-emotional states, and linguistic disparities (King and Ng, 2018). Admittedly, all these factors are vital for a successful L2 education, yet psycho-emotional factors and inner feelings appear to play a more crucial role in teaching and learning (Sikma, 2021). This shift toward emotions emerged by a new trend in psychology, called positive psychology (PP) that capitalized on positive emotions including love, joy, optimism, resilience, care, credibility, engagement, clarity, immediacy, rapport, passion, and so forth in pushing one forward in life and career (MacIntyre and Mercer, 2014; Gregersen and MacIntyre, 2021; Xie and Derakhshan, 2021). Instead of negative emotions (e.g., stress, anxiety), the proponents of PP argue for the power of positive emotions in generating happiness, wellbeing, and success (MacIntyre et al., 2019). An influential but limitedly explored positive emotion that can affect teachers’ pedagogical practices is hope which is a mental state by which the person sets clear goals, sustains motivation, and tries different paths to tackle setbacks, and expects to have a positive future (Snyder et al., 1991). Teaching L2 includes many challenges, complications, and adversities that are constantly slowing teachers’ didactic ship (Ghadyani et al., 2020) against which English as a foreign language (EFL) teachers must be hopeful, positive, and tough. With hope, teachers can achieve many outcomes in academia including wellbeing, optimism, job satisfaction, self-efficacy, and success (Alarcon et al., 2013; Ong et al., 2018).

Furthermore, EFL teachers’ hope may have a role to play in shaping and enhancing trust among teachers, students, principals, and organizations. Trust is one of the most essential elements of a well-functioning instruction and work (Tschannen-Moran and Hoy, 1998; Khany and Tazik, 2016). It refers to one’s willingness to be vulnerable to another person/group based on the confidence that he/she has in the intentions or behavior of that person/group (Hoy and Tschannen-Moran, 2003). It is an interpersonal and context-bound state that forms the cornerstone of education, engagement, and success (Hoy and Tarter, 2004). It has been found to influence classroom rapport, fairness, justice, wellbeing, work engagement, satisfaction, psychological empowerment, and task accomplishment (Tschannen-Moran and Hoy, 1998; Hoy and Tarter, 2004; Khany and Tazik, 2016; Hongwidjojo et al., 2018). Moreover, it can be argued that both hope and trust can contribute to and pave the way for EFL teachers’ sense of grit. Grit is a spiritual construct in educational psychology that resembles intrinsic motivation (Duckworth and Quinn, 2009). It concerns one’s enthusiasm and short-term perseverance for achieving goals by working hard despite challenges (Duckworth et al., 2010). In this regard, it is similar to the construct of resilience which has an influential role in PP. However, grit concerns one’s sustained effort toward a particular endeavor despite setbacks and adversities, while resilience is the ability to bounce back from those difficulties (Duckworth, 2016; Sikma, 2021).

Grit provides energy for teachers to work studiously to meet goals and develop personally (Duckworth, 2016). Academic grit, hence, can lead to success, interest, and efficiency in education (Keegan, 2017). Given its significance, in the past decades, grit has witnessed a surge of scholarly attention signifying its role in predicting various learner-related variables such as enjoyment, commitment, academic competence, wellbeing, resilience, achievement, enthusiasm, and satisfaction (Strayhorn, 2014; Jin and Kim, 2017; Reed and Jeremiah, 2017; Hodge et al., 2018; Steinmayr et al., 2018; Moen and Olsen, 2020). Nevertheless, the impact of academic grit on teacher-related factors and constructs has been mostly overlooked. Inspired by this gap, this mini-review article aimed to present a theoretical analysis of EFL teachers’ hope, trust, grit, and the way they can inform and shape one another. In so doing, theoretical underpinnings, conceptualizations, definitions, dimensions, and related empirical studies are provided to support the arguments.

## Background

### The Concept of Hope: Definitions and Theoretical Underpinnings

The concept of hope does not have many definitions in educational psychology, yet in their breakthrough research Snyder et al. (1991) described it as the process wherein the person thinks about his/her goals with determination and motivation and utilizes different strategies and ways to obtain such goals. Hope is a dynamic construct encompassing three components, namely, “goal,” “agency,” and “pathways” by which one determines goals, preserves his determination, and tries various tools to manage the difficulties in achieving those goals (Snyder, 2002). To put it differently, hope is a motivational inner state pertaining to one’s ability to develop pathways to desired goals, inspire oneself through agency, and employ those pathways (Rand and Cheavens, 2009).

Theoretically, hope is supported by Snyder’s (2000) hope theory that includes three components of goals, pathways, and freedom of choice (agency). Snyder regarded goals as the basis of hope theory because much of human behavior is goal-oriented. In hope theory, goals are verbal/visual representations that guide one’s action. Later, Snyder (2002) classified human goals into “approach goals” (i.e., positive goals one wants to achieve) and “avoidance goals” (negative things one wants to escape). According to this theory, pathways are alternative routes toward one’s desired future targets. The final constituent of this theory is agency or the capacity to maintain one’s motivation and effort when using a specific pathway. It resembles self-efficacy in that they both reflect one’s perceived ability to do something effectively. While self-efficacy is perception-oriented, agency is intention-oriented (Rand and Cheavens, 2009).

Another theory behind hope is self-determination theory (SDT) introduced by Deci and Ryan (1985). SDT is motivation theory that draws on agentic behavior in human (Adams et al., 2017). It has three components of *competence*, *autonomy*, and *relatedness* as psychological needs to be fulfilled. SDT is linked to hope in that it highlights agentic actions and causal agency by identifying pathways that assist one to satisfy his/her desires and get involved in self-direction and self-regulation to manage challenges and chances (Marques and Lopez, 2018). Therefore, it clearly echoes the three components of hope.

### Hope and Educational Outcomes

Due to its strong impact on one’s motivation and commitment, hope can bring about many positive outcomes in academic and organizational settings (Huang et al., 2019). An extensive body of research shows that hope can predict and be related to academic achievement, confidence, self-esteem, performance, resilience, success, positive mood, buoyancy, psychological wellbeing, satisfaction career behavior, cognitive ability, self-efficacy, and engagement (Hirschi, 2014; Feldman and Kubota, 2015; Gallagher et al., 2017; Dong et al., 2021). Despite these outstanding studies that approve the power of hope in education, little has been written about the role of hope in L2 education. Furthermore, the concept has been neither operationally defined nor statistically tested in SLA (Ghadyani et al., 2020). Consequently, there are still many unexplored avenues for L2 researchers to made scholarly attempts to unpack the effect of hope on EFL teachers’ and students’ behaviors and practices in the class. Given the significance of hope in education and its role in facilitating many academic domains (Dixson, 2019), further investigations are required to reveal the relationship between hope and other psycho-emotional variables in L2 education. One of such areas is the constitution of trust in the academic context. When the level of hope is high among EFL teachers, their degree of confidence in receiving positive intentions, feedbacks, and behaviors from students, colleagues, and principals enhances as well.

### Trust in Academic Arena

Trust is one of the most important components of a successful education (Hoy and Tarter, 2004). It is a multi-dimensional construct that must be perceived in relation to different stakeholders in academia (Khany and Tazik, 2016). However, in the case of teachers, trust pertains to colleagues and principals. Trust in colleagues is represented in teachers’ reliance on each other, while trust in principals concerns their tendency to keep promises and act in tune with the teachers’ interests. In a context full of trust, the quality of teaching, learning, task engagement, and justice improves exponentially (Hoy and Tarter, 2004). When the stakeholders have trust in each other, their cooperation to consistency to achieve long-term goals raises, as well. Moreover, it can be contended that trust is a fundamental element for students’ learning in that the establishment of this feeling facilitates the ground for the emergence of other positive academic outcomes like achievement, rapport, classroom engagement (Bergan, 2014). EFL teachers are also identified to psychologically empower in a setting which cares for trust among the workforce (Khany and Tazik, 2016). Many other outcomes are yet to be scientifically disclosed in L2 education.

### The Typology and Dimensions of Trust

**FIGURE 1:**
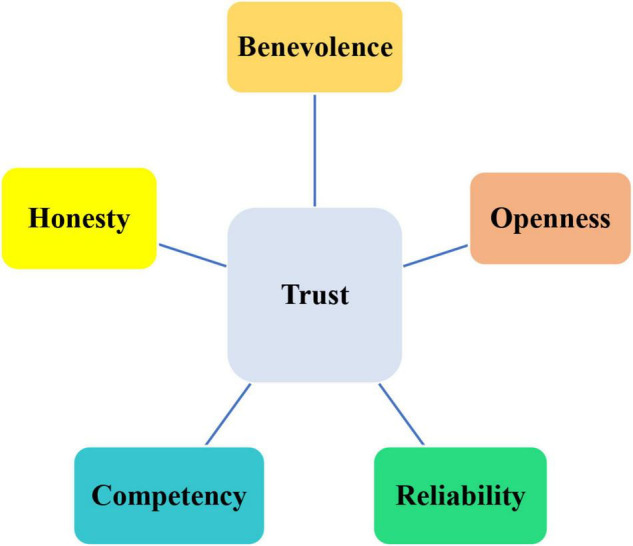
The dimensions of trust (Hoy and Tschannen-Moran, 2003, p. 185).

In the literature, different typologies and dimensions have been proposed for the construct of trust. It has been maintained by different scholars that trust is of three types; *relational* (depends on the relations between the trusting person and the other), *calculative* (depends on the others’ past behavior in the form of rewards and penalties), and *organic* (depends on shared beliefs and moral visions) (Rousseau et al., 1998; Solomon and Flores, 2001; Forsyth et al., 2006). Considering its dimensions, Hoy and Tschannen-Moran (2003) argued that trust includes five dimensions of *benevolence* (be sure that one’s wellbeing is secured by the trusted person/group), *reliability* (how much one can count on the other party), *competency* (the level of knowledge and skill in the trusted party), *honesty* (the character, truthfulness, and genuineness of the other party), and *openness* (not withholding information from the other party) as illustrated in in Figure 1.

In order to create a positive atmosphere laden with trust, teachers and other educational stakeholders must consider all these dimensions as each of these five dimensions play a significant role in teaching and learning process. None of them should be sacrificed for the other ones. When EFL teachers make sure of the kindness, frankness, honesty, expertise, and trustworthiness of their students, colleagues, and principals, they pedagogically perform better and more optimal outcomes will appear.

### The Definition of Academic Grit

As one of the most important cores of education (Yang, 2021), grit has been dismissed as a non-cognitive construct that causes people to work diligently and peruse their long-term wishes and goals (Bashant, 2014). It emanates from one’s enthusiasm, appetite, and perseverance to achieve pre-defined long-term goals in spite of adversities (Duckworth and Quinn, 2009). In contrast to Credé et al. (2017) who considered grit as a concept comparable to conscientiousness, Duckworth and Quinn (2009) saw it as a personality trait beyond motivation. While motivation is dynamic and context-bound, grit is quite constant across contexts and highlights long-term intellectual strengths, patience, resilience, and determination (Reed et al., 2013). As pinpointed by Duckworth et al. (2010), grit has two key components; (1) *perseverance in effort* (one’s effort and passion for fulfilling his/her goals despite challenges) and (2) *persistence of interests* (the consistency of one’s passion for achieving a goal).

### Academic Grit and L2 Education

In L2 education which involves many complexities and challenges, the concept of grit is a pivotal issue (Keegan, 2017). Now, L2 teachers and practitioners worldwide focus on the development of students’ competency and skills without dwelling too much on insignificant information and performances (Horn, 2013). Correspondingly, EFL teachers should try to encourage and cultivate students’ practical skills and perseverance in order to obtain their long-term goals (Taşpinar and Külekçi, 2018). Other than these skills, the goal of L2 education must be generating gritty teachers and students who do not fringe in the face of challenges and adversities common in L2 education (Duckworth et al., 2010). Grit is a critical construct in L2 education because its incorporation into academia can cause success and many other desired outcomes (Keegan, 2017). When EFL teachers and students are gritty, they perform better and manage the setbacks efficiently even after the instruction. In simple terms, they become long-lasting problem-solvers (Taşpinar and Külekçi, 2018). L2 education is full of hard times and frustrating difficulties that demand teachers be tough, resilient, hopeful, and gritty to survive such challenges with determination and eventually yield high performance (Keegan, 2017). Furthermore, focusing on grit development provides teachers and students with constant energy, self-discipline, perseverance, motivation, and self-control (Duckworth and Gross, 2014).

## Empirical Studies

In support of the variables of concern in this mini-review article, there are a number of empirical studies showing the impact of each variable on L2 education processes. Concerning hope in L2 contexts, research signifies that it is correlated with achievement, performance, self-esteem, career behavior, satisfaction, cognitive ability, success, resilience, confidence, self-efficacy, wellbeing, and engagement (Hirschi, 2014; Feldman and Kubota, 2015; Gallagher et al., 2017). Likewise, trust in L2 settings has been found to influence teachers’ professional learning, fairness, wellbeing, engagement, job satisfaction, psychological empowerment, and classroom rapport (Hoy and Tarter, 2004; Khany and Tazik, 2016; Hongwidjojo et al., 2018; Bellibaş and Gümüş, 2021). Both hope and trust can play a significant role in the development of grit in that when EFL teachers are hopeful and have trust in their colleagues and organizations, they manage the encountering setbacks better and become more gritty. The construct of grit itself has been identified to predict students’ and teachers’ enjoyment, success, commitment, wellbeing, resilience, enthusiasm, competence, achievement, interest, and satisfaction (Strayhorn, 2014; Jin and Kim, 2017; Keegan, 2017; Reed and Jeremiah, 2017; Hodge et al., 2018; Steinmayr et al., 2018; Moen and Olsen, 2020; Yang, 2021). Although these studies are promising, they have been simple one-shot, correlational works of research and the way EFL teachers’ hope and trust can influence their sense of grit still needs more research. Moreover, the dynamism and teachability of these variables have not been analyzed in teacher education. Consequently, the present mini-review sparked initial lights on the possible linkage of these three variables and called for more research in this domain.

## Concluding Remarks

In this mini-review it was contended that EFL teachers’ hope and trust can influence their sense of grit when facing L2 challenges. It was also stated that establishing a trusting academic milieu can pave the way for the growth of hope and grit among EFL teachers. The rational behind such claims is that when EFL teachers are hopeful about their career and the future, they look on the bright side when they encounter difficulties and complications. This, in turn, enhances their willingness to establish an academic environment that is based on trust among stakeholders. Consequently, in a social context in which the person is hopeful and has trust in colleagues and institutions, the management of adversities or being gritty is considerably easier than a place full of hopelessness, mistrust, and doubt. Hence, this study can be beneficial for EFL teachers, teacher trainers, principals, and L2 researchers. EFL teachers can use the ideas to improve their knowledge of emotions and psychological variables and their role in L2 education. They can also realize the value of having trust in a teaching context to generate success, hope, and grit. Teacher trainers may find this article valuable by using it as a guide to propose training courses and workshops in which the way teacher’ psycho-emotional factors can determine L2 education process are taught. They can educate teachers how to develop their own resilience and grit by practical methods and activities. Moreover, this study can be helpful for principals in that they can realize the importance of establishing trust in academia from which many positive academic outcomes can emerge. Finally, L2 researchers may find this mini-review beneficial in that they can run similar studies on hope, trust, and grit to fill the existing gaps in this area. For instance, they can use mixed-method deigns to gain richer data on the way these variables develop and function in L2 education. Most of the existing studies are correlational, hence future studies can use longitudinal designs to capture the developmental paths of these constructs. Additionally, the teachability of these variables through treatment courses can also be an interesting topic for research. Furthermore, the role of culture and interpersonal communication skills in the development of EFL teachers’ hope, trust, and grit is recommended to future scholars. Additionally, gathering triangulated data on the interplay of these three variables is also a fresh idea for research. Finally, the predictive power of hope and trust in relation to many other PP constructs as well as negative variables is also recommended.

## Author Contributions

YX took responsibility of research design and writing, contributed to the article, and approved the submitted version.

## Conflict of Interest

The author declares that the research was conducted in the absence of any commercial or financial relationships that could be construed as a potential conflict of interest.

## Publisher’s Note

All claims expressed in this article are solely those of the authors and do not necessarily represent those of their affiliated organizations, or those of the publisher, the editors and the reviewers. Any product that may be evaluated in this article, or claim that may be made by its manufacturer, is not guaranteed or endorsed by the publisher.

## References

[B1] Adams N. Little T. D. Ryan R. M. (2017). *Self-Determination Theory.* Dordrecht: Springer.

[B2] Alarcon G. M. Bowling A. Khazon S. (2013). Great expectations: a meta-analytic examination of optimism and hope. *Pers. Individ. Dif.* 54 821–827. 10.1016/j.paid.2012.12.004

[B3] Bashant J. (2014). Developing grit in our students: Why grit is such a desirable trait, and practical strategies for teachers and schools. *J. Leadersh. Instr.* 13 14–17.

[B4] Bellibaş M. Ş. Gümüş S. (2021). The effect of learning-centered leadership and teacher trust on teacher professional learning: evidence from a centralized education system. *Prof. Dev. Educ.* 1–26. 10.1080/19415257.2021.187923 [Epub ahead of print].

[B5] Bergan G. K. (2014). *Teacher Trust Levels and How They Differ Between School Settings and Impact Teacher Involvement in Student Achievement Activities.* Ph.D. dissertation. Kalamazoo, MI: Western Michigan University.

[B6] Credé M. Tynan M. Harms P. (2017). Much ado about grit: a meta-analytic synthesis of the grit literature. *J. Pers. Soc. Psychol.* 113 492–511. 10.1037/pspp0000102 27845531

[B7] Deci E. L. Ryan R. M. (1985). *Intrinsic Motivation and Self-Determination in Human Behavior.* New York, NY: Plenum.

[B8] Dixson D. D. (2019). Hope into action: how clusters of hope relate to success-oriented behavior in school. *Psychol. Sch.* 56 1493–1511. 10.1002/pits.22299

[B9] Dong H. Li W. Ye D. (2021). The influence of english as a foreign language teachers’ positive mood and hope on their academic buoyancy: a theoretical review. *Front. Psychol.* 12:801435. 10.3389/fpsyg.2021.801435 35058860 PMC8763973

[B10] Duckworth A. (2016). *Grit: The Power of Passion and Perseverance.* New York, NY: Simon and Schuster.

[B11] Duckworth A. Gross J. (2014). Self-control and grit: related but separable determinants of success. *Curr. Dir. Psychol. Sci.* 23 319–325. 10.1177/0963721414541462 26855479 PMC4737958

[B12] Duckworth A. L. Kirby T. A. Tsukayama E. Berstein H. Ericsson K. (2010). Deliberate practice spells success: Why grittier competitors triumph at the National Spelling Bee. *Soc. Psychol. Pers. Sci.* 2 174–181. 10.1177/1948550610385872

[B13] Duckworth A. L. Quinn P. D. (2009). Development and validation of the Short Grit Scale (Grit-S). *J. Pers. Assess.* 91 166–174. 10.1080/00223890802634290 19205937

[B14] Feldman D. B. Kubota M. (2015). Hope, self-efficacy, optimism, and academic achievement: distinguishing constructs and levels of specificity in predicting college grade-point average. *Learn. Individ. Differ.* 37 210–216. 10.1016/j.lindif.2014.11.022

[B15] Forsyth P. B. Barnes L. B. Adams C. M. (2006). Trust-effectiveness patterns in schools. *J. Educ. Adm.* 44 122–141.

[B16] Gallagher M. W. Marques S. C. Lopez S. J. (2017). Hope and the academic trajectory of college students. *J. Happiness Stud.* 18 341–352. 10.1007/s10902-016-9727-z

[B17] Ghadyani F. Tahririan M. H. Afzali K. (2020). Conceptualization of hope for EFL teaching within the Iranian context: a grounded theoretical model. *Issues Lang. Teach.* 9 27–58. 10.22054/ilt.2020.54699.531

[B18] Gregersen T. MacIntyre P. D. (2021). “Acting locally to integrate positive psychology and peace: practical applications for language teaching and learning,” in *Peace-Building in Language Education*, eds Oxford R. Olivero M. M. Harrison M. Gregersen T. (Bristol: Multilingual Matters), 177–195.

[B19] Hirschi A. (2014). Hope as a resource for self-directed career management: Investigating mediating effects on proactive career behaviors and life and job satisfaction. *J. Happiness Stud.* 15 1495–1512. 10.1007/s10902-013-9488-x

[B20] Hodge B. Wright B. Bennett P. (2018). The role of grit in determining engagement and academic outcomes for university students. *Res. High. Educ.* 59 448–460. 10.1007/s11162-017-9474-y

[B21] Hongwidjojo M. P. Monika M. Wijaya E. (2018). Relation of student-teacher trust with school well-being to high school students. *Psikodimensia* 17 162–167. 10.24167/psidim.v17i2.1664

[B22] Horn M. (2013). Building motivation, instilling grit: the necessity of mastery-based, digital learning. *Leadership* 1–2.

[B23] Hoy W. K. Tarter C. J. (2004). Organizational justice in schools: no justice without trust. *Int. J. Educ. Manage.* 18 250–259.

[B24] Hoy W. K. Tschannen-Moran M. (2003). “The conceptualization and measurement of faculty trust in schools: the omnibus T-Scale,” in *Studies in Leading and Organizing Schools*, eds Hoy W. K. Miskel C. G. (Greenwich, CT: Information Age Publishing), 181–208.

[B25] Huang T. Y. Souitaris V. Barsade S. G. (2019). Which matters more? Group fear versus hope in entrepreneurial escalation of commitment. *Strateg. Manage. J.* 40 1852–1881. 10.1002/smj.3051

[B26] Jin B. Kim J. (2017). Grit, basic needs satisfaction, and subjective well-being. *J. Individ. Differ.* 38 29–35. 10.1027/1614-0001/a000219

[B27] Keegan K. (2017). Identifying and building grit in language learners. *Engl. Teach. Forum* 55 2–9. 10.1016/j.jsurg.2020.03.021 32305336

[B28] Khany R. Tazik K. (2016). On the relationship between psychological empowerment, trust, and Iranian EFL teachers’ job satisfaction: the case of secondary school teachers. *J. Career Assess.* 24 112–129. 10.1177/1069072714565362

[B29] King J. Ng K.-Y. S. (2018). “Teacher emotions and the emotional labour of second language teaching,” in *Language Teacher Psychology*, eds Mercer S. Kostoulas A. (Bristol: Multilingual Matters), 141–157.

[B30] MacIntyre P. D. Gregersen T. Mercer S. (2019). Setting an agenda for positive psychology in SLA: theory, practice, and research. *Mod. Lang. J.* 103 262–274. 10.1111/modl.12544

[B31] MacIntyre P. D. Mercer S. (2014). Introducing positive psychology to SLA. *Stud. Second Lang. Learn. Teach.* 4 153–172. 10.14746/ssllt.2014.4.2.2

[B32] Marques S. C. Lopez S. J. (2018). “Promoting hope in children,” in *The Oxford Handbook of Hope*, eds Gallagher M. W. Lopez S. J. (New York, NY: Oxford University Press), 117–131.

[B33] Mercer S. (2020). The wellbeing of language teachers in the private sector: an ecological perspective. *Lang. Teach. Res.* 1 1–24. 10.1177/1362168820973510

[B34] Moen F. Olsen M. (2020). Grit: a unique protective factor of coaches well-being and burnout? *New Ideas Psychol.* 59:100794. 10.1016/j.newideapsych.2020.100794

[B35] Ong A. D. Standiford T. Deshpande S. (2018). “Hope and stress resilience,” in *The Oxford Handbook of Hope*, eds Gallagher M. W. Lopez S. J. (New York, NY: Oxford University Press), 1–96.

[B36] Rand K. L. Cheavens J. S. (2009). “Hope theory,” in *The Oxford Handbook of Hope*, eds Gallagher M. W. Lopez S. J. (New York, NY: Oxford University Press), 323–333.

[B37] Reed J. Pritschet B. L. Cutton D. M. (2013). Grit, conscientiousness, and the trans-theoretical model of change for exercise behavior. *J. Health Psychol.* 18 612–619. 22904153 10.1177/1359105312451866

[B38] Reed L. Jeremiah J. (2017). Student grit as an important ingredient for academic and personal success. *Dev. Bus. Simul. Exp. Learn.* 44 252–256.

[B39] Rousseau D. M. Sitkin S. B. Burt R. S. Camerer C. (1998). Not so different after all: a cross-discipline view of trust. *Acad. Manage. Rev.* 23 393–404.

[B40] Sikma L. (2021). “Building resilience: using BRiTE with beginning teachers in the United States,” in *Cultivating Teacher Resilience*, ed. Mansfield C. F. (Singapore: Springer), 85–101.

[B41] Snyder C. R. (2000). *Handbook of Hope: Theory, Measures, and Applications*. San Diego, CA: Academic Press.

[B42] Snyder C. R. (2002). Hope theory: rainbows in the mind. *Psychol. Inq.* 13 249–275. 10.1207/s15327965pli1304_01

[B43] Snyder C. R. Irving L. Anderson J. R. (1991). “Hope and health: measuring the will and the ways,” in *Handbook of Social and Clinical Psychology: The Health Perspective*, eds Snyder C. R. Forsyth D. R. (Elmsford, NY: Pergamon), 285–305.

[B44] Solomon R. C. Flores F. (2001). *Building Trust in Business, Politics, Relationships, and Life.* New York, NY: Oxford University Press.

[B45] Steinmayr R. Heyder A. Naumburg C. Michels J. Wirthwein L. (2018). School-related and individual predictors of subjective well-being and academic achievement. *Front. Psychol.* 9:2631. 10.3389/fpsyg.2018.02631 PMC6308923 30622497

[B46] Strayhorn T. L. (2014). What role does grit play in academic success of black male collegians at predominately white institutions? *J. Afr. Am. Stud.* 18 1–10.

[B47] Taşpinar K. Külekçi G. (2018). Grit: an essential ingredient of success in the EFL classroom. *Int. J. Lang. Educ. Teach.* 6 208–226. 10.18298/ijlet.3137

[B48] Tschannen-Moran M. Hoy W. (1998). Trust in schools: a conceptual and empirical analysis. *J. Educ. Adm.* 36 334–352.

[B49] Xie F. Derakhshan A. (2021). A conceptual review of positive teacher interpersonal communication behaviors in the instructional context. *Front. Psychol.* 12:2623. 10.3389/fpsyg.2021.708490 34335424 PMC8319622

[B50] Yang P. (2021). Exploring the relationship between Chinese EFL students’ grit, well-being, and classroom enjoyment. *Front. Psychol.* 12:762945. 10.3389/fpsyg.2021.762945 34777167 PMC8586070

